# Deep neural model with self-training for scientific keyphrase extraction

**DOI:** 10.1371/journal.pone.0232547

**Published:** 2020-05-15

**Authors:** Xun Zhu, Chen Lyu, Donghong Ji, Han Liao, Fei Li

**Affiliations:** 1 Key Laboratory of Aerospace Information Security and Trusted Computing, Ministry of Education, School of Cyber Science and Engineering, Wuhan University, Wuhan, Hubei, China; 2 School of Mathematics and Computer Science, Jianghan University, Wuhan, Hubei, China; 3 Laboratory of Language and Artificial Intelligence, Guangdong University of Foreign Studies, Guangzhou, Guangdong, China; 4 Collaborative Innovation Center for Language Research and Services, Guangdong University of Foreign Studies, Guangzhou, Guangdong, China; National University of Singapore, SINGAPORE

## Abstract

Scientific information extraction is a crucial step for understanding scientific publications. In this paper, we focus on scientific keyphrase extraction, which aims to identify keyphrases from scientific articles and classify them into predefined categories. We present a neural network based approach for this task, which employs the bidirectional long short-memory (LSTM) to represent the sentences in the article. On top of the bidirectional LSTM layer in our neural model, conditional random field (CRF) is used to predict the label sequence for the whole sentence. Considering the expensive annotated data for supervised learning methods, we introduce self-training method into our neural model to leverage the unlabeled articles. Experimental results on the ScienceIE corpus and ACL keyphrase corpus show that our neural model achieves promising performance without any hand-designed features and external knowledge resources. Furthermore, it efficiently incorporates the unlabeled data and achieve competitive performance compared with previous state-of-the-art systems.

## Introduction

With the explosive increase of scientific publications, it is important for users to better understand the key ideas of the articles. Keyphrases are usually regarded as phrases that represent the salient concepts of a document [1], and provide users with valuable information. The scientific keyphrases identification and classification is motivated by the increasing demand for efficiently finding relevant scientific publications and automatically understanding the key information of those publications, and it has received much academic interest over the past years [2–6]. Furthermore, it is also an important prerequisite task for downstream applications, such as summarization, information retrieval and question-answering.

**Fig 1:**
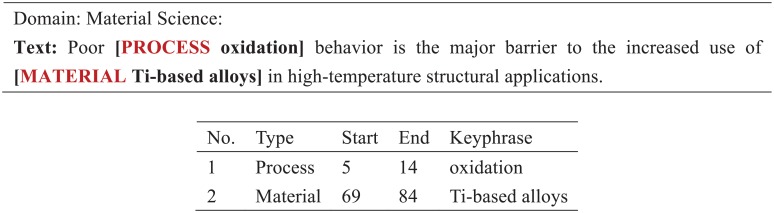
Keyphrases annotated in the ScienceIE corpus.

Scientific information extraction, including keyphrase and semantic relation extraction, is a curial step for understanding scientific publications. SemEval 2017 has organized a shared task on scientific information extraction (ScienceIE) [6]. The benchmark dataset consists of scientific articles in the Computer Science, Material Sciences and Physics domains, and the keyphrases in this dataset are annotated with three categories: TASK, PROCESS and MATERIAL. One annotated example in this dataset is illustrated in Fig 1. Gupta and Manning (2011) [7] introduced a human-annotated dataset containing abstracts from the ACL anthology. The keyphrases with three categories (DOMAIN, TECHNIQUE and FOCUS) are annotated in this corpus. These annotated datasets allow us to employ supervised machine learning methods for scientific keyphrase extraction.

Most studies have been conducted on keyphrase extraction [8]. On one hand, unsupervised methods, such as TF-IDF [9] based ranking method, achieve comparable performance in this task [10, 11]. On the other hand, various supervised learning methods, such as support vector machines (SVMs), are used to identify keyphrases [6, 12]. However, these systems need to pay much attention on feature engineering efforts.

Recently, deep learning has been widely used in natural language processing (NLP) [13–16], and it brings hope to reduce manual feature engineering in various tasks. Compare with hand-designed features and traditional discrete feature representation, it provides a different way to automatically learn dense features representation for text, such as words, phrases and sentences. Our method follows this line and builds the neural model based on the bidirectional long short-memory (LSTM) and conditional random field (CRF). The input word representation of our model is computed based on the word embeddings, part-of-speech (POS) embeddings and dependency embeddings.

However, the success of these supervised learning methods relies on large amounts of annotated data. The number of training instances of this task is limited and it is expensive to annotate much more data for supervised training of neural network models. As we know, there are massive unannotated scientific publications, which are publicly available. In this paper, we introduce self-training methods to the neural model to take advantage of these unlabeled scientific articles. We first train the model with the original training data, and label the unannotated text using this model. The high confidence data with the predicted label is selected into the training set. Then we retrain the model using the new training data. The self-training method repeatedly perform the above process, and a number of data is selected into the training set to improve the performance.

We evaluate our models on the SemEval 2017 ScienceIE corpus and ACL keyphrase corpus. Standard evaluation demonstrates that our neural model can achieve promising performance for scientific keyphrase extraction without any hand-designed features and external knowledge resources. In addition, our model with self-training method can efficiently utilize unlabeled data, and achieve competitive performance compared with other state-of-the-art systems.

## Related work

### Keyphrase extraction

Previous works on keyphrase extraction usually focus on documents in different domains, including news [17], scientific [4], meeting transcripts [10] and web text [18, 19]. The methods used for keyphrase extraction fall into two lines: supervised learning and unsupervised learning.

In the supervised learning research line, keyphrase extraction is formalized as a classification problem. These works first extract candidate phrases using some heuristic rules, and then train a classification model to predict whether a candidate phrase is a keyphrase or not. Different features have been used for this task [2, 20–22], including frequency features (e.g. TF-IDF), position features, structural features, syntactic features and external resource-based features.

In the unsupervised learning research line, it is usually formalized as a ranking problem. The kayphrases are usually ranked based on the TF-IDF [10, 23, 24] and term informativeness [25]. Besides the frequency information, more statistic and context information [26] has shown the importance in this task. Graph-based ranking is also widely used in unsupervised methods [17, 27]. It aims to build a graph and rank its nodes, which represent candidate keyphrases, according to their importance. Following this approach, topic information [28, 29], semantic information from knowledge bases [30, 31] and pretrained word embeddings [32, 33] have been incorporated into the graph-based ranking model to improve the performance.

Due to the lack of human-annotated corpus, previous work on scientific information extraction is limited. Gupta and Manning (2011) [7] introduced a dataset of scientific abstracts annotated with the three categories. Pattern-based bootstrapping approach is used to automatically extract these keyphrases. Tsai *et al*. (2013) [34] incorporated hand-designed features into unsupervised bootstrapping framework to improve the performance.

Scientific information extraction has attracted much attention in recent years, and it becomes the focus of SemEval 2017 Task10. This task includes three subtasks: keyphrase identification, keyhprase classification and relation extraction between keyphrases. It releases the ScienceIE dataset, and provides a benchmark to evaluate the system performance on this task. In this paper, we focus on kephrase extraction, including keyphrase identification and classification. Similar to named entity recognition (NER), we formalize keyphrase extraction as a sequence labelling problem.

### Deep learning

Deep neural networks, especially recurrent neural networks (RNN) [35] and LSTM-RNN [36] have been successfully used for the sequence labelling task. Collobert *et al*. (2011) [13] proposed a unified neural network framework, and performed various sequence labelling tasks, including POS-tagging, NER and chunking, simultaneously. Huang *et al*. (2015) [37] used hand-crafted features with LSTMs to improve the NER performance. The bidirectional LSTM-RNN combined with CRFs have been widely used in NER [37–40], and achieve promising performance.

Word2Vec [41] and GloVe [42] are effective algorithms for learning word representations to capture syntactic and semantic features for words. Recently, pre-trained language models with context, such as ELMo [43], BERT [44] and XLNet [45], have shown great power in the semantic representation of text, and achieved excellent performance in various NLP applications.

In scientific keyphrase extraction subtask of SemEval 2017 Task 10, top three systems all used RNN-based methods [46, 47]. On the top of the RNN layer, CRFs [48], which jointly model the label sequence, performed better compared to the softmax layer. Alzaidy *et al*. (2019) [49] explored a Bi-LSTM-CRF neural model, that captures long distance semantic information, for keyphrase extraction from scientific articles. Our work is related to the line and introduces self-training method to the neural model to leverage unlabeled scientific articles.

## Methods

### Label schemes for NER

We introduce the label scheme used for this task in this section. Following previous works on NER, we use the BILUO label scheme, which has been widely used for NER, for our task in this paper.

**Fig 2:**
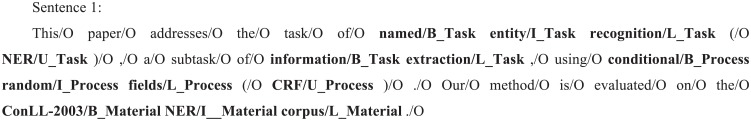
One example using the BILUO label scheme. Sentence 1 comes from the ScienceIE corpus.

B and L are used to label the beginning and end word of the keyphrase. U indicates the one-word keyphrase. I and O are used to label the the inside and outside words of the keyphrase. Meanwhile, suffix is added to represent the category of the keyphrase, such as PROCESS, MATERIAL and TASK. One example using the BILUO label scheme is shown in Fig 2.

### BLSTM-CRF model

**Fig 3:**
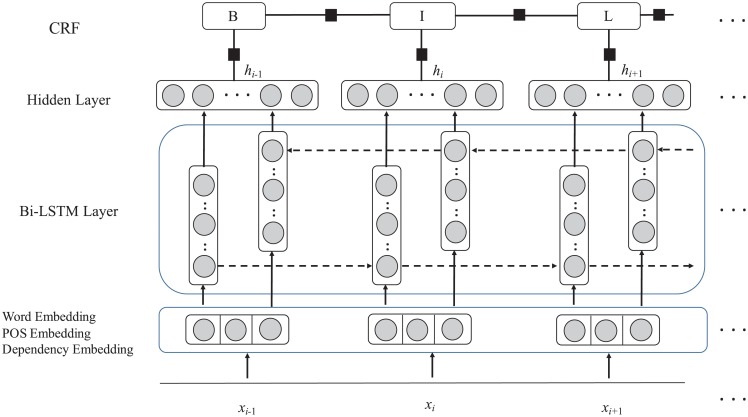
Overview of the BLSTM-CRF model.

Our method utilizes the bidirectional LSTM (BLSTM) and CRF for scientific keyphrase extraction. Fig 3 illustrates the BLSTM-CRF model used for this task. It first gets the word representation by concatenating word embeddings, POS embeddings and dependency embeddings. Then the Bi-LSTM layer takes the word representation as input and generate more complex features for the input sentence. Finally, CRF is added to predict the label sequence for the sentence. The BILUO label scheme mentioned in the above section is used to form the label sequence.

Word embeddings, which are trained from large scale raw corpus, can capture semantic information of the word. They are commonly used in the neural network models and give a continuous feature vector to represent the word. Given an input sentence *s* = {*w*_1_, *w*_2_, …, *w*_*n*_}, the word embedding lookup table function is used to get the word embedding *e*_*i*_ for each word *w*_*i*_.

Not only the word itself, but also the POS information and the dependency information of the word helps to extract keyphrases from the text. Similar to the word embeddings, the model uses the POS embedding and dependency embedding lookup table functions to get the POS representation *p*_*i*_ and dependency representation *d*_*i*_ for each word *w*_*i*_, respectively. Based on these embeddings, we get the word representation *x*_*i*_ = *e*_*i*_⊕*p*_*i*_⊕*d*_*i*_ for each word *w*_*i*_.

Then the input representation {*x*_1_, *x*_2_, …, *x*_*n*_} is fed into the Bi-LSTM layer, and the LSTM layer outputs more complex feature representations for the input sequences. The LSTMs perform well at capturing long range information, and bidirectional LSTM is introduced to incorporate the past and future information in the sequence data.

Formally, the left LSTM generates the feature representation $\vec{h_{i}}$ for each *x*_*i*_ by processing the input representation {*x*_1_, *x*_2_, …, *x*_*n*_} from left to right. In the similar way, the right LSTM generates the feature representation $\overset{\leftarrow }{h_{i}}$ from right to left. Then we obtain the final feature representation *h*_*i*_ through the hidden layer:

$$\begin{array}{c} h_{i}=tanh\left(W_{1}\left[\vec{h_{i}}⊕\overset{\leftarrow }{h_{i}}\right]+b_{1}\right) \end{array}$$ (1)

where ⊕ denotes the concatenation between two vectors.

Finally, we get the feature representation *h* = {*h*_1_, *h*_2_, …, *h*_*n*_} for the sentence *s*, use the CRF to predict the label sequence *y*.

The input sentence *s* = {*w*_1_, *w*_2_, …, *w*_*n*_} corresponds to a matrix *Z* ∈ *R*^*n*×*l*^, where *n* is the size of the input sequence, and *l* is the number of labels. Given sentence *s*, *Z*_*i*,*j*_ denotes the score of the word *w*_*i*_ with the *j*-th label. The score of an output sequence *y* is defined by:

$$\begin{array}{c} S\left(s,y\right)=\sum_{i=0}^{n}\left(Z_{i,y_{i}}+T_{y_{i-1},y_{i}}\right) \end{array}$$ (2)

where *y*_*i*_ is the label for the word *w*_*i*_, and $T_{y_{i-1},y_{i}}$ represents the transition score from the label *y*_*i*−1_ to *y*_*i*_.

Then the conditional probability of the sequence *y* is defined by:

$$\begin{array}{c} P\left(y|s\right)=\frac{exp(S(s,y))}{\sum_{y^{{}^{\prime }}\in Y\left(s\right)}exp\left(S\left(s,y^{{}^{\prime }}\right)\right)} \end{array}$$ (3)

where *Y*(*s*) is the set which contains all the possible label sequences of the sentence *s*.

Given the sentence *s*, the decoding process aims to find the label sequence with the highest score in *Y*(*s*).

### Self-training method

Most keyphrase extraction systems employ supervised machine learning method and achieve promising performance. Since the number of training instances in this task is limited and annotating more data is expensive, semi-supervised learning methods, which can utilize the annotated data and unannotated data, provide a possible way to improve the performance. To take advantage of the unannotated data, we apply self-training method to the neural model. The details of our method is illustrated in Algorithm 1.

**Algorithm 1** Self-training Method

**Input**: Training Set *L*, Unlabeled Data *U*, Confidence Set *C*, Probability Threshold *p*

**Output**: Model Parameters Θ

1 Parameters Θ Initialization

2 **for** *i* = 1 … *T* **do**

3  Training the model *model*_*i*_ using training set *L*

4  Predict the unlabeled data set *U* using *model*_*i*_

5  **for** each instance *s* in *U* **do**

6   **if** *P*(*y*|*s*)>*p* **then**

7    *C* ← *e*

8   **end if**

9  **end for**

10  *U* = *U* − *C*

11  *L* = *L*∪*C*_*l*_; *C*_*l*_ is the labelled set using *Model*_*i*_

12  *C* = *ϕ*

13 **end for**

14 **return** Model Parameters Θ

The basis of the self-training method is the BLSTM-CRF model. The model parameters and the training process in Line 3 are the same as the neural model. In each iteration, we first train the model using the training set, and then select the confidence set from the unlabeled data according to the probability of the instance. The probability is given by the trained model using the Eq 3. The instance with the probability higher than the threshold *p* is added into the confidence set. The selected confidence set with the label given by the trained model is add to training set, and the new training set will be used in the next iteration.

As described in Algorithm 1, the selection of the confidence set *C* is the key factor that affect the performance of self-training method, and how to measure the confidence of a tagging sequence is crucial to our method. Despite the conditional probability *P*(*y*|*s*) described in Eq 3, we further investigate the following two metrics to measure the confidence of the tagging sequence *y*:

$$\begin{array}{c} Confidence_{1}\left(y|s\right)=P\left(y|s\right)*N \end{array}$$ (4)

$$\begin{array}{c} Confidence_{2}\left(y|s\right)=\sqrt[{N}]{P(y|s)} \end{array}$$ (5)

where *N* is the length of sentence *s* and *P*(*y*|*s*) is the conditional probability of the sequence *y*.

### Training

The BLSTM-CRF model consists of the neural network layers and the CRF layer, and we will explain the training details of BLSTM-CRF network.

Given the training set ${\left\{x_{i},y_{i}\right\}}_{i=1}^{M}$, the max likelihood training objective is formally by

$$\begin{array}{c} S\left(\Theta \right)=\frac{1}{M}\sum_{i=1}^{M}logP\left(y_{i}|x_{i}\right)+\frac{\lambda }{2}∥\Theta {∥}^{2} \end{array}$$ (6)

where *x*_*i*_ is an input sentence and *y*_*i*_ is the corresponding golden label sequence. *P*(*y*_*i*_|*x*_*i*_) is the probability of the golden label sequence as defied in Eq 3. λ is the regularization parameter and Θ denotes all model parameters of the model.

AdaGrad [50] algorithm is employed to update the parameters of our model.

## Experiments

### Experimental settings

#### Data and evaluation

We evaluated scientific keyphrase extraction task on two publicly available datasets: ScienceIE corpus provided by SemEval 2017 Task 10 (https://scienceie.github.io/) and ACL keyphrase corpus (https://nlp.stanford.edu/pubs/FTDDataset_v1.txt). The ScienceIE corpus consists of 500 scientific paragraphs, which come from three different domains. These paragraphs are annotated with keyphrase in three categories: TASK, PROCESS, MATERIAL. The dataset is split into three parts, namely 350 notes for training, 50 notes for development and 100 notes for testing. On the other hand, the ACL keyphrase corpus consists of 462 scientific abstracts, and the human-annotated keyphrases in this corpus contain three categories: DOMAIN, TECHNIQUE and FOCUS. We split this dataset into three parts, namely 370 abstracts for training, 46 abstracts for development and 46 abstracts for testing. Table 1 shows the statistics of the two datasets used in the experiments.

**Table 1:** Statistics of the datasets.

	Training	Dev	Test
ScienceIE			
Sentences	2403	399	851
Keyphrases	6721	1154	2051
ACL			
Sentences	2159	283	272
Keyphrases	2999	389	392

According to the source and domains of the ScienceIE dataset, we crawled the unannotated scientific articles in the same three domains (CS, MC and Ph) from ScienceDirect (https://www.sciencedirect.com/) for self-training method. We randomly select 24,030 abstracts as the unannotated dataset from the website, and this dataset contains 156,459 sentences. For the ACL keyphrase corpus, we use the document collection from the ACL Anthology dataset as the unannotated dataset (https://acl-arc.comp.nus.edu.sg/archives/acl-arc-090501d1/data/txt/) [51]. This dataset contains 7,586 abstracts and 47,718 sentences.

Commonly used precision (P), recall (R) and F1 are used as evaluation metrics.

#### Pre-processing

Since our neural model takes the word embeddings, POS embeddings and dependency embeddings as the input representation, we use the Stanford CoreNLP library [52] to preprocess the raw text in the corpus, and get the tokenization, POS-tagging and dependency parsing information. Our neural models are implemented based on the LibN3L [53] package.

#### Post-processing

Many keyphrases, especially their first letters, often appear in the uppercase form in scientific articles, and the uppercase features helps to recognize the keyphrases. To improve the performance of our neural model, we apply a simple heuristic rule to the output of the neural model.

Our neural model outputs the keyphrases in the document, along with their categories and indexes. If one keyphrase contains the capital letter, we label all this phrase occur in the document as the keyphrase.

#### Hyper-parameter settings

We have tuned the hyper-parameters in our neural models on the development set. The hyper-parameters mainly include two parts. One is the structure definition of the neural network, including the size of different embeddings and the size of each hidden layer. The other part includes the hyper-parameters used in the training process.

We use GLOVE word embeddings [42] for our word embeddings initialization, and the dimension of word embeddings is 300. POS embeddings and dependency embeddings are randomly initialized and their dimension is set to 25. These randomly initialized embeddings are fine-tuned in the training process of our model, while the pre-trained word embeddings are not fine-tuned in our experiments.

To alleviate the overfitting problem in the training process, the dropout [54] method is applied to our neural model. When the dropout method is used, the F1 score of our model is improved by 5.9% on ScienceIE corpus and 3.6% on ACL corpus. Table 2 lists the details of these hyper-parameters.

**Table 2:** Hyper-parameter settings.

Type	Hyper-parameter
probability threshold *p*	0.8
Initial learning rate	0.01
Regularization parameter	10^−8^
dropout rate	0.4
Dim(emb(word))	300
Dim(emb(POS)), Dim(emb(DEP))	25
Hidden layer size	100

#### Baselines

The following baselines are used for system comparison in our experiments:

- **BLSTM-CRF**: The bidirectional LSTM combined with CRFs have been successfully used in the sequence labelling task, and achieve promising performance. Alzaidy *et al*. (2019) utilized this model and their results showed that it substantially outperformed some strong baselines and previous methods for keyphrase extraction.
- **BERT**: BERT is designed to pretrain deep bidirectional representations by jointly conditioning on both left and right context [44]. It has achieved state-of-the-art results in various NLP applications, including NER. Our BERT-based keyphrase extraction system is implemented based on an open source project NER-BERT-pytorch (https://github.com/lemonhu/NER-BERT-pytorch). The code of our BERT-based baseline is available at https://github.com/RingoTC/BERT-NER-ScienceIE.

### Results

#### Effects of the heuristic rule

Table 3 shows the performance of BLSTM-CRF model with and without the heuristic rule. BLSTM-CRF-H represents the BLSTM-CRF model using the heuristic rule.

**Table 3:** Effects of the heuristic rule.

Models	ScienceIE (P/R/F1)	ACL (P/R/F1)
BLSTM-CRF	47.4/40.5/43.7	40.7/31.4/35.4
BLSTM-CRF-H	47.3/43.1/45.1	39.9/31.4/35.1

Applying the heuristic rule to the output of our BLSTM-CRF model improves the F1 score from 43.7% to 45.1% on the ScienceIE corpus. It indicates that the heuristic rule helps the model to recognize more keyphrases. Especially, the use of this rule helps to improve the recall of the model. It improves the recall of the neural model from 40.5% to 43.0%, while their precisions are almost the same (47.3% vs 47.4%). This is reasonable since tokens with uppercase letters are often proper nouns or abbreviations for specific terms. These words are often keyphrases in the document.

However, this heuristic rule does not help to improve the performance on the ACL corpus. The likely reason is that the ACL test set contains less keyphrases than the ScienceIE corpus, and there are few cases that satisfy this heuristic rule. Thus, we will not apply the heuristic rule to the BLSTM-CRF model on the ACL corpus in the following experiments.

#### Self-training method

Furthermore, we introduce self-training method to the neural model to leverage the unannotated data. It selects instances with high confidence from the unlabeled data set, and adds them into the training set.

Despite the conditional probability *P*(*y*|*s*), different confidence functions described in Eqs 4 and 5 are investigated in the experiments. The baseline neural model used for the ScienceIE corpus is the BLSTM-CRF model with the heuristic rule, while the BLSTM-CRF model without the heuristic rule is used for the ACL corpus.

When different confidence functions are used in the self-training method, their F1 scores are very close. According to the results listed in Table 4, we choose the conditional probability *P*(*y*|*s*) as the confidence function for the ScienceIE corpus, and $\sqrt[{N}]{P(y|s)}$ is used for the ACL corpus.

**Table 4:** Effects of the self-training method.

Models	ScienceIE (P/R/F1)	ACL (P/R/F1)
Baseline	47.3/43.1/45.1	40.7/31.4/35.4
*P*(*y*|*s*)	49.2/42.6/45.7	42.6/30.6/35.6
*P*(*y*|*s*)*N	48.6/43.2/45.7	45.0/29.6/35.7
$\sqrt[{N}]{P(y|s)}$	48.6/42.9/45.6	45.4/31.6/37.3

The increase of the training data helps to extract scientific keyphrases. When the self-training method is applied to the BLSTM-CRF model using heuristic rule, it improves the F1 score from 45.1% to 45.7% on the ScienceIE corpus. Considering the ACL corpus, it improves the F1 score of the BLSTM-CRF model from 35.4% to 37.3%.

#### Effects of embeddings

Considering the input layer of our neural model, it gets the input word representation by concatenating word embeddings, POS embeddings and dependency embeddings. To study the effects of POS and dependency embedding, we conduct ablation test to show the impact of these embeddings. The baseline model is the BLSTM-CRF model. Table 5 shows the results of the ablation test on the ScienceIE and ACL corpus.

**Table 5:** Ablation test for different embeddings.

Models	ScienceIE (P/R/F1)	ACL (P/R/F1)
BLSTM-CRF	47.4/40.5/43.7	40.7/31.4/35.4
No Pos embeddings	48.2/39.2/43.2	40.9/29.3/34.2
No dependency embeddings	47.6/39.9/43.4	41.5/28.6/33.8

From the Table 5, we can see that both POS embedding and dependency embeddings contribute to the scientific keyphrase extraction. Compared with the model without these embeddings, the system performs slightly better on the ScienceIE corpus. Consider the ACL corpus, the BLSTM-CRF model outperforms the model without the POS or dependency embeddings, with the improvement of 1.2% and 1.6%, respectively.

In addition to the word information itself, incorporating POS and dependency information into the BLSTM-CRF model has the potential to improve the performance. For example, BLSTM model can recognize “[main/JJ corrosion/NN products/NNS]” as the Material keyphrase, while it can not extract this keyphrase without its POS information. Its POS information helps to improve the performance of the BLSTM-CRF model.

#### Comparison with previous systems

Tables 6 and 7 show the results of our model on the ScienceIE and ACL corpus respectively, together with previous state-of-the-art performance systems for comparison.

**Table 6:** Results of our model on the ScienceIE corpus, together with other top-performance systems.

Models	F1(%)
Gupta [7]	9.8
Tsai [34]	11.9
AI2 [46]	44
Luan *et al*. (2017) [55]	46.6
BLSTM-CRF	43.7
BERT	35.1
**SL-BLSTM-CRF-H**	**45.7**

**Table 7:** Results of our model on the ACL corpus, together with other top-performance systems.

Models	F1(%)
BLSTM-CRF	35.4
BERT	31.6
**SL-BLSTM-CRF**	**37.3**

Gupta [7] and Tsai [34] are unsupervised learning methods. Gupta uses pattern-based bootstrapping approach for automatic keyphrase extraction, and Tsai incorporates hand-designed features into unsupervised bootstrapping framework. AI2 [46] is the best system participating in SemEval 2017 scientific keyphrase extraction task. It employs neural networks and CRFs for this task, and adds scientific terms from external resources as features. Luan *et al*. (2017) [55] has achieved the best keyphrase extraction performance on ScienceIE corpus, and it introduces inductive and transductive semi-supervised learning to the neural tagging models.

From the Table 6, we can see that the performance of the supervised learning and semi-supervised learning methods is much higher than the performance of the unsupervised learning methods on the ScienceIe corpus. The human-annotated data allows the use of supervised learning and semi-supervised learning methods, and helps to improve the scientific keyphrase extraction task.

Among the 17 teams participating the SemEval 2017 scientific keyphrase extraction subtask, we find that the BLSTM-CRF based neural networks achieve top performance in this task. The F1 score of the BLSTM-CRF model used in this paper is slightly lower than that of AI2, which is the best system participating in SemEval 2017 ScienceIE task. The likely reason is that our baseline model performs keyphrase extraction without any external knowledge resources and feature engineering.

Our SL-BLSTM-CRF-H model achieves competitive performance compared with the best performance systems on the ScienceIE corpus. It achieves 2% and 10.6% improvements of F1 score over BLSTM-CRF and BERT, respectively. This demonstrates the effectiveness of our Bi-LSTM-CRF for this task and the importance of character representation. Considering the ACL keyphrase corpus, our best model SL-BLSTM-CRF outperforms these strong baselines, with the improvements of 1.9% and 5.7% over BLSTM-CRF and BERT, respectively.

## Conclusions

In this paper, we represented the neural network based model for recognizing and classifying the keyphrases from scientific documents. We employed the state-of-the-art BLSTM-CRF model for scientific keyphrase extraction. Self-training method, which can leverage the unannotated data, was applied to the neural model to improve the performance.

Experiments on the ScienceIE and ACL keyphrase datasets demonstrated the effectiveness of our models. Without any external knowledge resources and manually feature engineering, our BLSTM-CRF model achieved comparable performance. When applying self-training method to the BLSTM-CRF model, it further improved the performance and achieved competitive performance compared with previous state-of-the-art systems.
